# Circulating microRNAs and Plasma Gelsolin as Biomarkers of Sepsis: Molecular Insights and Prospects for Precision Medicine

**DOI:** 10.3390/biom15111621

**Published:** 2025-11-18

**Authors:** Mircea Stoian, Leonard Azamfirei, Sergio Rares Bandila, Adina Stoian, Dragoș-Florin Babă, Claudia Bănescu

**Affiliations:** 1 Department of Anesthesiology and Intensive Care Medicine, George Emil Palade University of Medicine, Pharmacy, Science and Technology of Târgu Mureș, 540103 Târgu Mureș, Romania; mircea.stoian@umfst.ro (M.S.); leonard.azamfirei@umfst.ro (L.A.); 2 Orthopedic Surgery and Traumatology Service, Marina Baixa Hospital, Av. Alcade En Jaume Botella Mayor, 03570 Villajoyosa, Spain; sergiob1976@gmail.com; 3 Department of Pathophysiology, George Emil Palade University of Medicine, Pharmacy, Science and Technology of Târgu Mureș, 540136 Târgu Mureș, Romania; 4 Department of Cell and Molecular Biology, George Emil Palade University of Medicine, Pharmacy, Science and Technology of Targu Mureș, 540142 Targu Mureș, Romania; dragos-florin.baba@umfst.ro; 5 Genetics Department, Center for Advanced Medical and Pharmaceutical Research, George Emil Palade University of Medicine, Pharmacy, Science and Technology of Targu Mures, Gheorghe Marinescu Street No. 38, 540136 Targu Mureș, Romania; claudia.banescu@umfst.ro

**Keywords:** sepsis, biomarkers, plasma gelsolin, circulating *miRNAs*, artificial intelligence, personalized medicine

## Abstract

Sepsis is a major medical emergency, characterized by a dysfunctional immune response to infection, which often progresses to multiple organ failure and death. Early diagnosis and prognostic evaluation present significant challenges due to limitations in the specificity and sensitivity of traditional biomarkers. This narrative review summarizes recent evidence on the potential of circulating microRNAs (*miRNAs*) such as *miR-150*, *miR-146a*, *miR-223*, *miR-155*, *miR-122*, and *miR-4772-5p* and plasma gelsolin (pGSN) as diagnostic and prognostic markers in sepsis. We discuss mechanisms involved and their potential for integration with artificial intelligence (AI) in personalized medicine. PubMed, Embase, and Web of Science databases were searched for relevant literature. Original research, systematic reviews, and meta-analyses focused on the diagnostic or prognostic value of circulating *miRNAs* or pGSN in sepsis were included; opinion papers and case reports were excluded. Altered expression of certain circulating microRNAs correlates with disease severity and mortality. Among circulating microRNAs (*miRNAs*), miR-122 and miR-150 have become the most consistently validated biomarkers in clinical studies, associated with sepsis severity and death rates. Additionally, other *miRNAs* such as *miR-146a*, *miR-155*, and *miR-223* play roles in modulating immune and endothelial responses, highlighting the complex regulation of sepsis pathophysiology. Low pGSN concentrations at admission are associated with severe sepsis and acute respiratory distress syndrome, and serve as an independent predictor of mortality. Preclinical studies suggest that supplementation with exogenous pGSN could increase survival. AI algorithms show promising results for early sepsis detection and optimization of therapeutic decisions. However, combining circulating *miRNAs* and plasma gelsolin (pGSN) into AI-based models is still an exploratory idea that needs prospective validation, assay standardization, and multicenter studies before it can be used clinically.

## 1. Introduction

Sepsis is a life-threatening condition marked by a dysfunctional immune response to infection. It can advance to septic shock, multiple organ failure, and death, especially without early diagnosis and treatment [1]. Sepsis remains a significant challenge for global health systems and is the leading cause of death among critically ill patients. Intensive care admission and extensive medical resources are often required, particularly within the first 48 h after presentation, and mortality can reach up to 46.4% [2]. Additionally, the prolonged use of life support measures (such as invasive mechanical ventilation and venous, arterial, and urinary catheters) increases the risk of nosocomial infections, which are common complications in intensive care units [3,4]. The number of severe sepsis cases is expected to rise from 2.3 million in 2020 to 2.8 million by 2030, driven by an aging population, a higher burden of comorbidities, and increasing microbial resistance to antibiotics [5,6,7].

Although antimicrobial resistance (AMR) is not the primary focus of this review, it further highlights the urgent need for rapid and precise diagnostic biomarkers. Earlier identification of sepsis could reduce the unnecessary use of broad-spectrum antimicrobials, prevent treatment delays, and improve patient outcomes.

The pathophysiology of sepsis is complex, involving a network of interactions between the innate immune system, endothelium, coagulation, and cellular metabolism. Activation of Toll-like receptors (TLRs) by pathogens triggers an inflammatory cascade via the nuclear factor kappa-light-chain-enhancer of activated B cells (NF-κB) pathway. This causes a “cytokine storm,” the massive release of pro-inflammatory cytokines, such as Tumor Necrosis Factor Alpha (TNF-α), interleukin (IL)-1β, and IL-6 [8,9].

Sepsis involves a complex and dysregulated immune response where pro-inflammatory and counteracting anti-inflammatory mechanisms happen at the same time, leading to endothelial dysfunction, blood clotting issues, and metabolic problems that contribute to multiple organ failure [8,10]. In this pathophysiological state, early detection of patients in the initial stages of sepsis is essential for improving outcomes.

Traditional biomarkers such as C-reactive protein (CRP) and procalcitonin (PCT) have been widely used for diagnosing and monitoring sepsis; however, their clinical performance remains limited. CRP indicates overall inflammation rather than infection-specificity, whereas PCT exhibits variable kinetics depending on the infectious source and the presence of organ dysfunction. Although these markers aid in early suspicion and antibiotic stewardship, their diagnostic accuracy and prognostic value remain limited, especially in differentiating sepsis from sterile inflammation or non-infectious organ failure [11]. Therefore, the search for novel, more specific, and dynamically regulated biomarkers, such as circulating microRNAs (miRNAs) and plasma gelsolin (pGSN), is of growing clinical and research interest. These short, non-coding RNA molecules are stable in biological fluids and accurately reflect the molecular disturbances of the immune and inflammatory response in sepsis [12,13].

*miRNAs* regulate various molecular pathways involved in sepsis, including those that trigger inflammatory responses, oxidative stress, apoptosis, and angiogenesis. Several studies have reported altered levels of *miRNAs* in sepsis patients, such as *miR-150*, *miR-146a*, and *miR-223*. These changes have been linked to disease severity, organ failure, and risk of death.

Given the major clinical impact of sepsis—and the complexity of the pathophysiological mechanisms involved—the early and accurate identification of high-risk patients is vital to reduce mortality, complications, and the economic burden associated with critical care [14]. The use of molecular biomarkers, especially *miRNAs*, not only offers the potential for early diagnosis and more precise risk stratification, but could also help guide therapeutic decision-making tailored to individual patient immune profiles [15,16].

Besides *microRNAs*, plasma gelsolin (pGSN) has emerged as a significant circulating biomarker of the host’s inflammatory and immune response in sepsis. pGSN is a multifunctional actin-binding protein involved in extracellular actin scavenging, endothelial stabilization, and modulation of leukocyte activation. Lower plasma levels of pGSN have been linked to increased disease severity, organ failure, and worse outcomes in septic patients [17,18].

In contrast, *miRNAs* regulate many of the same pathways at the post-transcriptional level, implying that pGSN and *miRNAs* could serve as complementary markers that reflect different yet interconnected aspects of the host response and tissue injury resolution. Among emerging molecular biomarkers, *microRNAs* (*miRNAs*) and plasma gelsolin (pGSN) have garnered increasing attention for their complementary roles in immune regulation and tissue injury during sepsis. *miRNAs* are small, non-coding RNAs that regulate gene expression post-transcriptionally, influencing inflammatory and immune signaling pathways [14,15]. Circulating miRNAs are stable and reliably detectable in plasma, making them promising diagnostic and prognostic biomarkers. Similarly, plasma gelsolin (pGSN) is an actin-binding protein with extracellular anti-inflammatory and cytoprotective properties; its levels decrease significantly during sepsis and correlate with disease severity and adverse outcomes [17,18]. Together, these molecules offer complementary insights into immune dysregulation and cellular damage, which are supported by their combined evaluation in this narrative review and precede the methodological overview.

## 2. Methods

This work was designed as a narrative, integrative review of the literature on the diagnostic and prognostic value of circulating *microRNAs* (*miRNAs*) and plasma gelsolin (pGSN) in sepsis and critical illness. The integrative review approach was chosen to synthesize findings from both clinical and experimental studies, combining molecular evidence with translational perspectives relevant to biomarker validation and clinical application.

A comprehensive search was conducted in PubMed, Embase, and Web of Science databases for studies published between 2008 and 2025, using the following keywords and Boolean combinations: “*microRNA*” OR “*miRNA*” OR “plasma gelsolin” AND “sepsis” OR “septic shock” OR “critical illness.” Only peer-reviewed articles published in English were included.

Additionally, the search strategy was expanded to include the literature on the use of artificial intelligence (AI), machine learning (ML), and computational modeling to combine and predict sepsis outcomes with miRNA and pGSN data. The following additional keywords were used: “artificial intelligence,” “machine learning,” “predictive model,” “biomarker integration,” and “data-driven diagnosis.” This ensured the inclusion of translational and computational research focused on AI-based biomarker modeling and multi-omics integration.

Studies were eligible if they reported experimental or clinical data on *miRNA* or pGSN expression in the context of sepsis, systemic inflammation, or organ dysfunction. Exclusion criteria included non-original publications (e.g., editorials, letters, conference abstracts), studies lacking biomarker quantification, and animal models unrelated to sepsis.

Data extraction covered the study population, the biomarker(s) assessed, the diagnostic criteria (Sepsis-2 or Sepsis-3), the severity scores, the main outcomes, and the biological mechanisms proposed. The quality assessment evaluated the study design, sample size, and biomarker validation methods.

## 3. Pathophysiology of Sepsis

### 3.1. Risk Factors and Susceptibility to Infections

**Figure 1:**
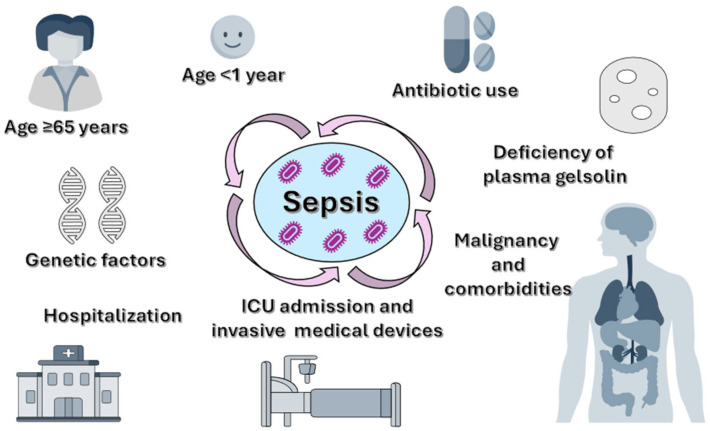
The clinical, demographic, and molecular factors associated with the development of severe sepsis (age extremes, comorbidities, extended hospitalization, antibiotic use, invasive medical devices, genetic predisposition, pGSN deficiency) and its progression to severe forms.

While even an apparently minor infection can cause sepsis, severe cases typically occur in vulnerable patients with weakened immune systems (Figure 1). Older patients (≥65 years) are among the most affected groups due to immunosenescence, decreased physiological reserves, and related comorbidities such as diabetes mellitus, chronic kidney failure, or chronic obstructive pulmonary disease [19,20,21].

Newborns and children under one year old are also highly vulnerable because their immune systems are still developing, limiting their ability to mount an effective response to infections. Additionally, both younger and older age groups face increased risks of hospitalization and exposure to invasive medical devices, which can lead to the development of nosocomial infections [22]. Other risk groups include pregnant women in the postpartum period (due to pregnancy-specific immune changes); patients with chronic conditions (such as diabetes, cancer, liver, kidney, or lung diseases); those with immunosuppression (congenital, iatrogenic, or acquired, including Human Immunodeficiency Virus (HIV), chemotherapy, or corticosteroids); and individuals who have undergone recent major surgeries or prolonged hospital stays [19,23]. Moreover, survivors of a previous episode of sepsis are at higher risk of recurrence and death during the first year after discharge, with nearly 50% requiring rehospitalization [19].

Prolonged intensive care unit (ICU) stays are linked to a higher risk of nosocomial infections due to exposure to invasive devices, such as venous and urinary catheters, parenteral nutrition, and mechanical ventilation [22].

Besides clinical factors, genetic variants have been identified that impact the immune response at the molecular level, increasing susceptibility to sepsis and its severe forms. Genetic polymorphisms in genes encoding pathogen recognition receptors, such as Toll-like receptors (TLR-4 or TLR-2), can change immune cell responses to bacterial lipopolysaccharides (LPS), influencing the development of the inflammatory response [24,25]. For instance, the TLR4 polymorphism Asp299Gly is associated with a hyporeactive response to LPS, leading to delayed and insufficient activation of innate immunity, thereby increasing the risk of severe sepsis [26].

Similarly, the variability of human leukocyte antigen (HLA) haplotypes involved in antigen presentation influences immune system recognition of pathogens, determining individual differences in susceptibility and disease severity [27,28].

Recent studies have identified rare mutations or altered expression levels in genes such as FER (Fps/Fes related tyrosine kinase) [29], Vacuolar Protein Sorting 13 Homolog A (VPS13A [30], and IRAK4 (Interleukin-1 Receptor-Associated Kinase 4) [31], which modulate essential inflammatory pathways, such as NF-kB or JAK/STAT (Janus kinase/Signal Transducers and Activators of Transcription). These changes influence the immunological phenotype and prognosis of patients [32]. Genome-wide association studies have helped decipher these genetic correlations and have supported the advancement of personalized medicine for sepsis [33].

FER is a cytoplasmic tyrosine kinase involved in maintaining vascular integrity, regulating cell adhesion, and coordinating innate immune responses. Recent genomic studies have identified a variant (rs4957796) significantly associated with 28-day survival in patients with pneumococcal sepsis [29], suggesting a protective role in regulating excessive inflammation and maintaining endothelial homeostasis [29,34,35]. Functionally, FER influences neutrophil chemotaxis and stabilizes the endothelial barrier, most likely through indirect regulation of the NF-kB signaling pathway [34]. Inactivation of FER in murine models led to unbalanced cytokine production, increased capillary permeability, and reduced responsiveness to bacterial endotoxins such as LPS [36].

### 3.2. Dysregulated Host Immune Response: Early Triggers and Signaling Pathways

**Figure 2:**
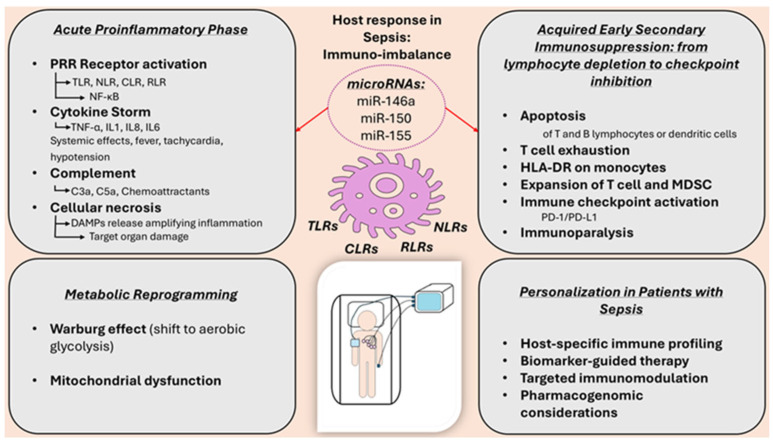
Immuno-inflammatory imbalance in sepsis: microbial and lesion signals activate Toll-like, C-type lectin, NOD-like, and RIG-I-like receptors, leading to NF-kB activation, cytokine release, and complement activation (C3a, C5a). These effects generate immunothrombosis and tissue damage. Simultaneously, early immunosuppression—characterized by lymphocytic apoptosis, decreased HLA-DR expression on monocytes, and activation of PD-1/PD-L1—and metabolic reprogramming (the Warburg effect and mitochondrial dysfunction) maintain the imbalance and contribute to organ failure. miRNA-146a, -155, and -150 modulate the response and can help guide personalized therapy.

Sepsis is characterized by complex systemic immune dysfunction, including early imbalances between proinflammatory hyperactivation and profound, progressive immunosuppression [37] (Figure 2). The early inflammatory response is triggered by the Pathogen-Associated Molecular Patterns (PAMPs) and endogenous products released from damaged cells—damage-associated molecular patterns (DAMPs)—via pattern-recognition receptors (PRRs), especially toll-Like Receptors (TLRs), NOD-like receptors (NLRs), and RIG-I-like receptors (RLRs) [38]. The activation of these pathways leads to the nuclear translocation of the transcription factor NF-kB and the initiation of the cytokine storm through the massive release of proinflammatory mediators (TNF-α, IL-1β, IL-6); this results in fever, tachycardia, and changes in tissue perfusion [39]. At the same time, inflammation activates the complement and coagulation systems, leading to immunothrombosis and cellular ischemia.

### 3.3. Endothelial Dysfunction, Immunothrombosis, and Sepsis-Related Coagulopathy

Systemic inflammation synergistically activates the complement and coagulation cascades (Figure 2). Anaphylatoxins C3a and C5a help recruit and activate leukocytes, while also stimulating parallel platelet aggregation and tissue factor expression on endothelial cell surfaces. This concurrent activation leads to the formation of intravascular microthrombi, a process called immunothrombosis, which impairs microcirculatory flow and contributes to tissue ischemia and cellular hypoxia [40]. Subsequent cell necrosis further amplifies the inflammatory response through the additional release of DAMPs [41].

### 3.4. Acquired Immunosuppression and Immunometabolic Reprogramming

Paradoxically, early inflammatory hyperactivation occurs in parallel with a state of immunosuppression, marked by widespread lymphocyte apoptosis, reduced expression of the human leukocyte antigen (HLA)-DR molecule on monocytes, and functional depletion of regulatory T lymphocytes and suppressor myeloid cells [42]. Additionally, the activation of certain immune pathways (Programmed Cell Death Protein-1 (PD-1)/Programmed Death-Ligand 1 (PD-L1)) exacerbates immune dysfunction and increases susceptibility to secondary infections [43].

An essential aspect of immune dysfunction in sepsis is the metabolic reprogramming of immune cells, which switch from oxidative phosphorylation to aerobic glycolysis (the Warburg effect) [44]. Simultaneous mitochondrial dysfunction, lactate buildup, and oxidative stress contribute to worsening organ damage and subsequent multiple organ failure.

## 4. Emerging Biomarkers in Sepsis

### 4.1. miRNAs: Epigenetic Regulators and Emerging Biomarkers in Sepsis

*miRNAs* are essential for the post-transcriptional regulation of gene expression. In sepsis, their expression profile is significantly changed, impacting both inflammatory hyperactivation and immune response suppression [44].

Figure 2 summarizes the dual inflammatory and immunosuppressive responses in sepsis, showing how dysregulated activation of innate immune pathways causes cytokine storm, immunothrombosis, and multi-organ damage.

For example, miR-146a inhibits TLR4 expression and reduces NF-κB activation, exerting an anti-inflammatory effect; meanwhile, *miR-155* mediates pro-inflammatory effects by stimulating the production of pro-inflammatory cytokines and worsening lung dysfunction [45,46].

Low *miR-150* levels are linked to poor prognosis, and *miR-122* signals early liver dysfunction. Other miRNAs, such as miR-223, have a protective role in lung inflammation. These epigenetic signatures support the use of *miRNAs* as biomarkers for immunological and prognostic stratification in sepsis [47]. For instance, low levels of miR-150 have been associated with increased mortality, making it a potential diagnostic and prognostic biomarker [48].

The pathophysiology of sepsis involves complex interactions between uncontrolled inflammation and progressive immunosuppression, with metabolic, genetic, and epigenetic factors playing a role. Understanding these integrated mechanisms is the necessary basis for developing personalized treatments and optimizing early interventions in critical care. Besides sepsis, *miRNAs* have also been extensively studied in other critical syndromes, such as acute respiratory distress syndrome (ARDS), acute pancreatitis, major trauma, and systemic inflammatory response syndrome (SIRS) [49]. For example, miR-155 contributes to the amplification of inflammation and LPS-induced lung injury in ARDS [50]. Conversely, a protective role has recently been confirmed for *miR-223* in models of post-traumatic or transfusion-induced lung injury [51].

In both trauma-induced and sepsis-associated acute respiratory distress syndrome (ARDS), *miR-223* plays a key role in regulating pulmonary inflammation. Elevated levels of miR-223 have been observed in bronchoalveolar lavage fluid from patients and experimental models of ARDS after severe trauma, transfusion, or infection [51].

In murine models, genetic removal of *miR-223* causes increased neutrophil infiltration and pulmonary edema, while administering *miR-223* externally decreases alveolar epithelial inflammation and reduces tissue damage. These results show that *miR-223* has a protective role in different ARDS causes—whether from trauma or sepsis—by lowering excessive inflammation and maintaining alveolar structure [13,52].

The specificity of the epigenetic response in sepsis—marked by the low expression of miR-150 and the increase of *miR-122*—reflects the imbalance between inflammation and immunosuppression and supports the utility of these biomarkers as immunological stratification tools [13,52]. The immune imbalance in sepsis results from a chaotic response that is simultaneously pro-inflammatory and immunosuppressive, producing an exaggerated inflammatory response that is worsened by metabolic and epigenetic disturbances. Understanding these integrative mechanisms provides the basis for personalized medicine in intensive care.

*miRNAs* consist of approximately 20–24 nucleotides and regulate gene expression through *mRNA* degradation and/or translational repression. *miRNAs* act by binding to target *mRNA* sequences via base pairing [15]. Through this mechanism, *miRNAs* precisely and specifically modulate fundamental biological processes such as inflammation, immune response, cellular metabolism, and apoptosis [53]. In sepsis, immune homeostasis is severely disrupted, and the expression of certain circulating *miRNAs* is profoundly altered, correlating with disease severity, organ dysfunction, and patient mortality. These molecules reflect a patient’s immunological status, and some directly influence key signaling pathways such as NF-kB, TLR4, Mitogen-Activated Protein Kinase (MAPK), or the systemic inflammatory response [52]. Recent studies also show that several *microRNAs* influence innate immune signaling beyond traditional NF-κB and TLR4 pathways. Specifically, *miR-223*, *miR-146a*, and *miR-155* have been linked to the regulation of cytosolic nucleic acid sensing and interferon signaling through the STING (Stimulator of Interferon Genes) pathway [54].

**Figure 3:**
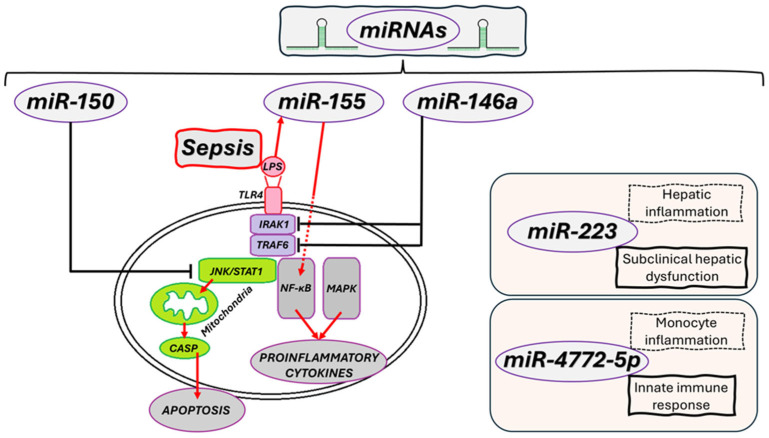
The Role of *miRNAs* in Sepsis Pathogenesis and Precision Medicine: MiRNAs regulate LPS-TLR4-IRAK1/TRAF6-NF-κB/MAPK (*miR-155* ↑, *miR-146a* ↓) pathways and have anti-inflammatory/antiapoptotic roles (miR-4772-5p (monocyte)), supporting their potential as biomarkers and for therapeutic stratification.

These data establish the empirical basis for the mechanistic models and molecular interactions discussed below and summarized in Figure 3 and Table 1.

By fine-tuning STING expression and downstream type I interferon responses, these miRNAs may affect the balance between antimicrobial defense and hyperinflammation in sepsis.

This emerging link underscores the complex interplay between epigenetic regulation and innate immune activation, suggesting that *miRNA*–STING interactions could serve as new therapeutic targets for immunomodulation in critical illness. For this reason, miRNAs are being increasingly studied as diagnostic biomarkers, predictors of severity and survival, and as potential targets for personalized therapies. A detailed overview of clinical and preclinical studies examining circulating microRNAs (*miRNAs*) and plasma gelsolin (pGSN) in sepsis is provided later in Table 2 and Table 3. 

#### 4.1.1. *miR-146a*—Negative Regulator of Inflammation

*miR-146a* plays a crucial role in inhibiting the TLR4/NF-kB pathway by suppressing the expression of tumor necrosis factor receptor-associated factor 6 (TRAF6) and Interleukin-1 Receptor-Associated Kinase 1 (IRAK1), two essential amplifiers of inflammatory signaling [55]. This mechanism results in a decrease in proinflammatory cytokine production (Figure 3), limiting the expansion of the immune response. In the early stages of sepsis, *miR-146a* overexpression has a protective effect, helping attenuate the excessive inflammatory response. However, in later phases, persistently elevated levels can promote a state of profound immunosuppression, impairing the host’s ability to fight secondary infection [56].

As a dynamic biomarker, serum *miR-146a* levels can indicate the shift from systemic inflammation to immunosuppression, offering an opportunity for early immunological stratification of patients [57]. Therapeutic mimetics of *miR-146a* have shown the ability to significantly reduce proinflammatory cytokine production in preclinical models, indicating their potential for translation [58]. Currently, *miR-146a* is primarily used as a circulating marker of immune response dynamics (from hyperinflammation to immunosuppression); its clinical application, on the other hand, remains unproven, and caution is advised due to the risk of immunosuppression [59].

#### 4.1.2. *miR-150*—Prognostic Biomarker and Anti-Inflammatory Regulator

*miR-150* is well-studied in the context of sepsis, known for its role in reducing inflammation and protecting endothelial tissues. Its prognostic value is supported by a clinical study involving 223 critically ill patients—including 138 with sepsis—which revealed low plasma levels of *miR-150* to be significantly linked to liver and kidney dysfunction, as well as long-term mortality, with predictive power comparable to the Acute Physiology and Chronic Health Evaluation (APACHE) score [59,60]. Although it does not have proven diagnostic value in distinguishing septic from non-septic patients, miR-150 modulates NF-kB1 expression, leading to the suppression of TNFα, IL-6, Intercellular Adhesion Molecule 1 (ICAM1), Vascular Cell Adhesion Molecule 1 (VCAM1), and E-selectin, resulting in anti-inflammatory and protective effects in endothelial cells such as Human Umbilical Vein Endothelial Cells (HUVEC) and supporting vascular barrier integrity (Figure 3) [15]. In animal models, overexpression of miR-150 reduced endoplasmic reticulum (ER) stress and improved cardiac function in septic cardiomyopathy, through Metastasis-Associated Lung Adenocarcinoma Transcript 1 (MALAT1)/NF-kB and Signal Transducer and Activator of Transcription 1 (STAT1)/c-Jun N-terminal Kinase (JNK)-dependent mechanisms [59]. Furthermore, overexpression of *miR-150-5p* in murine models of septic cardiomyopathy also showed protective effects, such as decreased apoptosis and enhanced cardiac function [59]. In human clinical trials, lower levels of *miR–150* have been directly associated with increased mortality risk in critically ill patients, indicating its potential as a reliable prognostic biomarker [61]. Currently, *miR-150* mainly has prognostic utility; low plasma levels correlate with adverse outcomes and organ dysfunction. Dynamic measurement at 0, 24, and 48 h from plasma via Reverse Transcription Quantitative Polymerase Chain Reaction/Digital PCR (RT-qPCR/Dpcr) is recommended, with integration of standard biomarkers and Sequential Organ Failure Assessment (SOFA) and APACHE II scores. Clinical application has not yet been demonstrated; main challenges include the targeting of delivery, determining the optimal timing of administration considering the hyperinflammation-immunosuppression transition, and safety concerns [62].

#### 4.1.3. *miR-155*—Inflammatory Response Enhancer

*miR-155* is a proinflammatory microRNA induced primarily by LPS exposure, involved in activating the NF-κB pathway and regulating the synthesis of TNF-α and IL-6 (Figure 3) [63]. Its expression steadily increases in severe forms of sepsis and correlates with endothelial dysfunction, extensive tissue damage, and the cytokine storm [64].

Pharmacological inhibition of *miR-155* in animal models has resulted in reduced systemic inflammation and improved survival [65,66]. In clinical context, *miR-155* has been proposed as an indicator of hyperinflammatory severity, but its therapeutic targeting remains at the preclinical stage [67].

Although experimental data suggest that modulation of *miRNA* expression could attenuate hyperinflammatory responses, these strategies remain at an early stage and have not yet been validated in human sepsis. Pharmacological inhibition with modified anti-*miR-155* locked nucleic acid has been shown to reduce inflammation and improve survival in preclinical models, but has not yet been clinically validated in humans [67,68,69]. Therefore, the therapeutic potential of *miRNAs* in sepsis should currently be viewed as experimental, pending further clinical research development.

#### 4.1.4. *miR-223*—Fine Regulator of Inflammatory Homeostasis

*miR-223* regulates the balance between inflammation activation and resolution, playing a role in controlling neutrophil recruitment and maintaining immune homeostasis. Although a defined clinical use is yet to be established, preclinical studies on models of ARDS and trauma suggest a protective role in lung inflammation and tissue damage prevention. Currently, *miR-223* is mainly used as a marker of an inflammatory lung phenotype (measured by serum or bronchoalveolar lavage (BAL) RT-qPCR, without a point-of-care test or standard clinical application (Figure 3). Pulmonary delivery of *miR-223* for lower alveolar inflammation, potentially inhibiting NOD-like Receptor Family Pyrin Domain Containing 3 (NLRP3), is a potential direction. At present, evidence is limited to preclinical studies, with no validation in humans [68].

#### 4.1.5. *miR-122* as a Biomarker of Hepatic Dysfunction

*miR-122* is mainly expressed in hepatocytes and plays a vital role in regulating lipid metabolism and liver inflammation. In sepsis, circulating levels rise significantly before typical changes detected by liver tests, indicating subclinical liver damage [69]. Due to this sensitivity, *miR-122* can serve as an early biomarker for liver dysfunction and enhance the prognostic profiling of critically ill patients (Figure 3) [70]. In sepsis, plasma *miR-122* increases early, predicting subclinical hepatic dysfunction; it is independently linked to 30-day mortality. Its inclusion could improve prognostic accuracy (through RT-qPCR/dPCR measurement, without the option for point-of-care testing) [71,72].

#### 4.1.6. *miR-4772-5p*—The Emerging Epigenetic Signature

*miR-4772-5p* has recently been recognized for its role in regulating monocyte activation and associated inflammatory pathways. Emerging studies show that its expression varies significantly between bacterial and fungal infections, as well as between patients with favorable and poor outcomes [73]. This variation highlights its potential as a marker for immunological stratification within sepsis, and as part of multi-*miRNA* signatures used to identify endophenotypes [74]. In sepsis, *miR-4772-5p* is an emerging marker for stratification: it increases in cluster of differentiation (CD)14 monocytes (including after TLR stimulation) and can differentiate SIRS from sepsis, specifically for bacterial and fungal infections. Its clinical application remains experimental (determination by RT-qPCR/dPCR). Currently, there are no validated targets or therapies, and the absence of a murine homologue limits preclinical evaluation [67].

Although promising diagnostic and prognostic correlations exist, most circulating *miRNAs* (including *miR-223* and *miR-4772-5p*) have only been validated in preclinical or retrospective human studies. Their measurement still depends on quantitative reverse-transcription PCR (RT-qPCR) or digital PCR (dPCR) conducted in centralized labs, with no point-of-care tests available for real-time clinical use [67,68,69].

The lack of standardized pre-analytical handling, extraction, normalization, and reference materials is a significant obstacle to the routine application of these processes. Developing harmonized protocols and calibration across laboratories will be essential to ensure reproducibility and comparability before moving forward with prospective clinical validation.

An overview of clinical and preclinical studies examining circulating microRNAs (*miRNAs*) and plasma gelsolin (pGSN) in sepsis is provided in Table 2 and Table 3. Table 2 summarizes human clinical research on the diagnostic and prognostic performance of circulating *miRNAs* (e.g., *miR-150*, *miR-122*) and pGSN, while Table 3 displays experimental and translational models that explore mechanistic pathways and therapeutic potential.

**Table 2:** Human studies investigating circulating microRNAs (miRNAs) and plasma gelsolin (pGSN) in sepsis and severe infection.

Study (Author, Year)	Biomarker(s)	Population/Setting	N (If Stated)	Sepsis Type/Diagnostic Criteria	Severity Scores Used	Organ System Focus	Outcomes (Direction)	Notes
Vasilescu et al., 2009 [49]	*miR-150* (plasma)	Adult ICU patients with severe sepsis and septic shock	30 sepsis + 20 healthy controls	Sepsis-2 criteria; infection confirmed microbiologically and ≥2 SIRS criteria	NR	Predominantly pulmonary and abdominal infections	↓ miR-150 levels significantly associated with ↑ mortality; inverse correlation with IL-18 and TNF-α	First clinical study identifying miR-150 as a prognostic plasma biomarker in sepsis; expression measured by RT-qPCR
Roderburg et al., 2013 [59]	*miR-150* (serum)	Critically ill adults; subset with sepsis	223 total patients (138 with sepsis) + 76 healthy controls	Sepsis diagnosed according to ACCP/SCCM (Sepsis-2) criteria	APACHE II and SOFA scores	Mixed etiologies —mainly abdominal and pulmonary sepsis	↓ miR-150 correlated with ↑ SOFA and APACHE II scores and higher mortality; low miR-150 = poor prognosis	Independent validation of miR-150 as a prognostic marker in critical illness and sepsis; confirmed inverse relation with inflammatory cytokines and organ failure scores
Ma et al., 2018 [61]	*miR-150*	Adult ICU patients diagnosed with sepsis; plasma samples analyzed and compared with healthy controls	40 sepsis + 30 healthy controls	Sepsis-3 criteria (infection + organ dysfunction)	SOFA	Systemic/vascular endothelial dysfunction	↓ miR-150 associated with ↑ mortality and ↑ inflammatory cytokines; in vitro overexpression suppressed LPS-induced NF-κB1 activation and endothelial apoptosis	Combined clinical + HUVEC experimental evidence showing miR-150 down-regulation contributes to sepsis-related endothelial injury and poor outcome
Zhou et al., 2024 [51]	*miR-223*, *miR-146a miR-155*	Patients with severe polytrauma; pulmonary tissue and plasma samples analyzed	24 trauma patients + control samples	systemic inflammatory response following polytrauma (sepsis-like immune dysregulation)	NR	Pulmonary/systemic inflammation	Combined C5 and CD14 inhibition induced an anti-inflammatory miRNA expression profile; ↑ miR-146a and ↓ miR-155 correlated with reduced cytokine release	Translational study linking trauma-related systemic inflammation and pulmonary miRNA regulation; supports mechanistic parallels with sepsis-induced lung injury and ARDS
Abou El-Khier et al., 2019 [73]	*miR-122* (plasma)	Adult ICU patients with sepsis compared to healthy controls	40 sepsis + 20 controls	Sepsis diagnosed according to Sepsis-2 (SIRS + infection criteria)	APACHE II	Hepatic dysfunction	↑ miR-122 levels in sepsis vs. controls; expression correlated positively with disease severity and mortality	Supports miR-122 as a diagnostic and prognostic biomarker reflecting liver dysfunction and systemic inflammation in sepsis
Self et al., 2019 [17]	Plasma gelsolin (pGSN)	Adults hospitalized with community-acquired pneumonia (CAP); prospective, multicenter cohort in U.S. hospitals	655 patients	CAP; subset fulfilling Sepsis-3 criteria analyzed	NR in manuscript	Respiratory/systemic	↓ pGSN at admission predicted higher risk of ICU admission, need for mechanical ventilation, and 30-day mortality	Demonstrated prognostic value of low plasma gelsolin as an early biomarker of severe outcomes in infection-related sepsis; supports link between pGSN depletion, inflammation, and poor prognosis

Legend: Summary of human clinical studies examining circulating *microRNAs* (*miRNAs*) and plasma gelsolin (pGSN) in sepsis and severe infection. The table details the study population, diagnostic criteria, severity scores, and clinical outcomes. Studies used either Sepsis-2 or Sepsis-3 criteria depending on publication year; severity was usually assessed with SOFA and/or APACHE II. NR in the manuscript indicates data not reported in the original publication. Abbreviations: miRNA—microRNA; pGSN—plasma gelsolin; SOFA—Sequential Organ Failure Assessment; APACHE II—Acute Physiology and Chronic Health Evaluation II; HUVEC—human umbilical vein endothelial cell; PSI—Pneumonia Severity Index; CURB-65—clinical score for CAP severity (confusion, urea > 7 mmol/L, respiratory rate ≥ 30/min, low blood pressure, age ≥ 65 years).

Most clinical studies examining circulating *miRNAs* in sepsis used the Sepsis-3 diagnostic criteria proposed by Singer et al. (2016) [1] and evaluated disease severity with the Sequential Organ Failure Assessment (SOFA) or APACHE II scoring systems.

The study populations mainly consisted of adult patients with sepsis of pulmonary or abdominal origin, often linked to Gram-negative bacterial infections (*Escherichia coli*, *Klebsiella pneumoniae*, *Pseudomonas aeruginosa*).

These clinical variables were considered in interpreting biomarker correlations, particularly the prognostic roles of *miR-122*, *miR-150*, and *miR-146a* in relation to organ dysfunction and outcome severity. Additionally, *miR-155*, although mainly studied in experimental CLP and LPS models, has consistently been linked to amplifying systemic inflammation through NF-κB and STAT3 activation, highlighting its importance in sepsis pathophysiology [1].

The consistent use of standardized diagnostic and scoring systems enhances the comparability of results across studies, even within the narrative synthesis approach of this review.

**Table 3:** Experimental and preclinical studies on circulating *microRNAs* (*miRNAs*) and plasma gelsolin (pGSN) in sepsis.

Study (Author, Year)	Biomarker(s)	Model/Species	Experimental Design	Organ System Focus	Key Findings
Feng et al., 2017 [68]	*miR-223*	Mouse model of mitochondrial DAMP–induced acute lung injury (ALI)	Sepsis lung inflammation induced by mitochondrial damage–associated molecular patterns (mtDAMP-induced ALI)	Lung (ARDS-like injury)	Neutrophil-derived miR-223 inhibits NLRP3 activation, reduces IL-1β release, and attenuates ALI (ARDS-like) injury.
An et al., 2018 [56]	*miR-146a*	Mouse model of LPS-induced sepsis and cardiomyocyte culture	Endotoxin-induced sepsis (LPS 10 mg/kg, i.p.)	Heart (septic cardiomyopathy)	miR-146a overexpression suppresses IRAK1/TRAF6, decreases NF-κB signaling, reduces myocardial injury, and improves function.
Cao et al., 2021 [66]	*miR-155*	Mouse model of cecal ligation and puncture (CLP)-induced sepsis	CLP-induced polymicrobial sepsis	Intestine/systemic inflammation	Improved barrier integrity; ↓ NF-κB activity; ↓ inflammation
Lee et al., 2007 [75]	pGSN	Mouse model of cecal ligation and puncture (CLP)–induced sepsis	CLP-induced polymicrobial sepsis and endotoxin challenge	Systemic/multi-organ	Demonstrated that pGSN depletion contributes to sepsis mortality; exogenous pGSN supplementation restored immune homeostasis and improved outcomes, suggesting therapeutic potential
Zhi et al., 2024 [39]	pGSN	Mouse model of SARS-CoV-2 spike protein–induced cytokine storm and acute lung injury (ALI)	Recombinant pGSN administration	Lung systemic	Demonstrated the anti-inflammatory and cytoprotective effects of exogenous gelsolin in cytokine storm and ALI; findings parallel therapeutic potential in sepsis-induced ARDS

Legend: Experimental and preclinical studies on miRNAs and plasma gelsolin (pGSN) in sepsis or ARDS-like injury. Models typically include CLP and LPS challenges or cytokine storm-induced ALI. Key findings highlight mechanistic pathways and the effects on outcomes. Abbreviations: CLP—cecal ligation and puncture; LPS—lipopolysaccharide; ARDS—acute respiratory distress syndrome; ALI—acute lung injury; pGSN—plasma gelsolin; miRNA—microRNA.

Collectively, these clinical and preclinical data support the translational importance of *miRAs* and pGSN as biomarkers that indicate immune dysregulation and tissue damage in sepsis, providing a conceptual link to the diagnostic and prognostic implications discussed in the next section.

Although circulating *miRNAs* show strong promise as immunoregulatory biomarkers and potential therapeutic targets, their clinical application is still limited by several technical and biological barriers. These include low in vivo stability and delivery efficiency of synthetic mimics or inhibitors, non-specific tissue distribution, and possible off-taret gene interactions that could disrupt normal signaling networks [67,68].

Moreover, immune responses to oligonucleotide-based therapies and the absence of standardized delivery systems—such as exosome-based or lipid nanoparticle methods—pose significant translational challenges. Consequently, *miRNA*-focused treatments for sepsis should currently be considered experimental and need further validation through multicenter prospective studies before they can be widely used in clinical settings [67,68].

### 4.2. Cytokine–miRNA Regulatory Axis in Sepsis: Roles of miRNA-509-3p, miRNA-145-5p and miRNA-494

A detailed overview of clinical and preclinical studies examining circulating microRNAs (*miRNAs*) and plasma gelsolin (pGSN) in sepsis is provided later in Table 2 and Table 3. These data establish the empirical foundation for the mechanistic models and molecular interactions discussed below and summarized in Figure 3 and Table 1.

Experimental studies have demonstrated that *miR-509-3p* and *miR-494* are transcriptionally activated by pro-inflammatory cytokines such as TNF-α and IL-1β, leading to the suppression of cystic fibrosis transmembrane conductance regulator (CFTR) *mRNA* and protein expression in epithelial cells [76,77]. CFTR down-regulation impairs epithelial ion and fluid transport, increases surface viscosity, and reduces the clearance of inflammatory mediators—mechanisms that, when activated in the lung during systemic inflammation, may contribute to alveolar fluid buildup, impaired gas exchange, and heightened susceptibility to secondary infections [78].

These molecular changes are not exclusive to genetic cystic fibrosis but can also happen as an acquired response in severe inflammation, including sepsis-induced lung injury (SILI) [79]. The cytokine–*miRNA*–CFTR pathway shows how systemic inflammation can cause local barrier dysfunction through post-transcriptional mechanisms.

Meanwhile, *miR-145-5p* plays a crucial regulatory role in vascular smooth muscle and endothelial cells, influencing cytoskeletal structure and intercellular junction proteins. Inflammatory cytokines like IL-6 and TGF-β have been shown to increase *miR-145-5p*, which in turn promotes endothelial contraction, actin stress-fiber formation, and increased permeability—features of microcirculatory dysfunction in sepsis [80].

These data collectively support the existence of a cytokine-driven *miRNA* regulatory network that affects both epithelial and endothelial compartments in sepsis. The induction of *miR-509-3p* and *miR-494* links inflammatory signaling to failure of epithelial ion transport, while *miR-145-5p* contributes to vascular leak and endothelial instability [76,77,78]. This integrated cytokine–*miRNA*–barrier axis provides a clear mechanism for sepsis-related organ dysfunction and highlights potential biomarkers and therapeutic targets for future translational research.

### 4.3. pGSN in Sepsis: Diagnostic and Prognostic Implications

Beyond established clinical and genetic factors, pGSN deficiency has emerged as a new risk factor for severe infections, sepsis, and ARDS. Although plasma gelsolin (pGSN) and circulating microRNAs (*miRNAs*) are different biomarker types, both indicate the severity and regulation of the host response in sepsis.

Several miRNAs involved in cytoskeletal remodeling and inflammation, such as *miR-21*, *miR-146a*, and *miR-155*, target signaling pathways that also regulate pGSN expression and actin dynamics.

Therefore, pGSN may act as a complementary indicator to *miRNA* profiles, offering insights into cellular injury, immune dysregulation, and endothelial dysfunction.

pGSN has multiple biological roles: it regulates inflammation, clears extracellular actin, stabilizes the endothelial barrier, and neutralizes bacterial toxins. In sepsis, pGSN levels drop sharply, reaching 25–30% of normal values within the first 6 h, a drop linked to extracellular actin presence and the exaggerated inflammatory response [39].

Preclinical studies in murine models using LPS and cecal ligation and puncture (CLP) techniques have demonstrated that exogenous pGSN supplementation boosts survival, reduces cytokine storms, and increases IL-10 levels, producing an anti-inflammatory effect.

Additionally, pGSN can bind and neutralize various bacterial endotoxins, including LPS, lipoteichoic acid, and platelet-activating factor. It also inhibits neutrophil and platelet activation and stabilizes the vascular barrier, reducing permeability and edema [39].

The therapeutic potential of pGSN has been demonstrated in Streptococcus pneumoniae models, where replenishing pGSN boosted Nitric Oxide Synthase 3 (NOS3)-mediated phagocytosis, lowered bacterial load, and significantly improved survival, even without antibiotics. In chemically induced ARDS models, pGSN administration lowered pulmonary leukocyte infiltration, alveolar permeability, and mortality, indicating a direct protective effect on the lungs [39].

In humans, low plasma pGSN levels at ICU admission have been linked to severe sepsis and ARDS, and serve as an independent predictor of mortality. Survivors showed a gradual recovery in pGSN levels, which correlated with clinical improvement.

In 2024, a Phase II study was launched to assess the safety and efficacy of recombinant human pGSN as an adjunctive treatment in patients with moderate to severe ARDS [81]. These data suggest that monitoring pGSN levels and administering the exogenous form [75] as therapy can play a crucial role in preventing severe progression and improving patient outcomes.

Taken together, circulating miRNAs and pGSN reflect complementary aspects of the host response in sepsis—the former regulating gene expression and signaling pathways, and the latter indicating cytoskeletal integrity and extracellular actin clearance.

This complementarity provides the foundation for integrative molecular profiling, which will be discussed in the next section.

## 5. Integrating *miRNA* and pGSN Profiles for Personalized Medicine in Sepsis

Advances in systems biology (multi-omics) demonstrate the integration of transcriptomic, proteomic, and epigenetic data including miRNA expression to define distinct molecular subtypes of sepsis (e.g., hyperinflammatory, immunosuppressive, or metabolically deregulated phenotypes) [73].

Such integrative approaches support a personalized approach to treating critically ill patients, where *miRNA* signatures could guide the selection of immunotherapies such as anti-PD-1, IL-7 or be used for monitoring treatment effectiveness, response, or early adjustments to life support strategies [53].

This strategy paves the way toward individualized management of critically ill patients, combining molecular markers like *miRNA* and pGSN with computational modeling and AI-based prediction to enhance diagnostic precision and prognostic accuracy [66].

## 6. Integrative Perspectives: Artificial Intelligence and Biomarker-Guided Sepsis Management

### 6.1. Early Diagnosis and Prognostic Stratification with Artificial Intelligence

Artificial intelligence (AI) algorithms, especially those based on machine learning (ML) and deep learning (DL), have shown significant potential for early sepsis detection and prognostic stratification. By processing large-scale data from electronic health records (EHRs), vital signs, and laboratory results, these models can identify subtle multidimensional patterns preceding clinical diagnosis, often outperforming traditional systems such as NEWS2 or qSOFA in sensitivity and timeliness [82].

The Targeted Real-time Early Warning System (TREWS), developed at Johns Hopkins, has been shown to predict sepsis onset up to six hours before clinical recognition, reducing mortality by approximately 20% in selected groups. Likewise, advanced recurrent neural network (RNN)-based architectures, such as Multi-task Gaussian Process (MGP)-RNNs and Sepsis Finger, achieved areas under the receiver operating characteristic curve (AUROC) values greater than 0.85 [83,84].

Despite these advances, current AI models rely almost exclusively on clinical and physiological variables. The integration of molecular and epigenetic biomarkers—particularly circulating microRNAs (*miRNAs*) and plasma gelsolin (pGSN)—remains conceptual and exploratory, without prospective clinical validation.

Such integration could theoretically improve immune phenotyping and early detection of high-risk subgroups, but it requires standardized molecular assays, harmonized data infrastructures, and regulatory validation before being used clinically.

**Figure 4:**
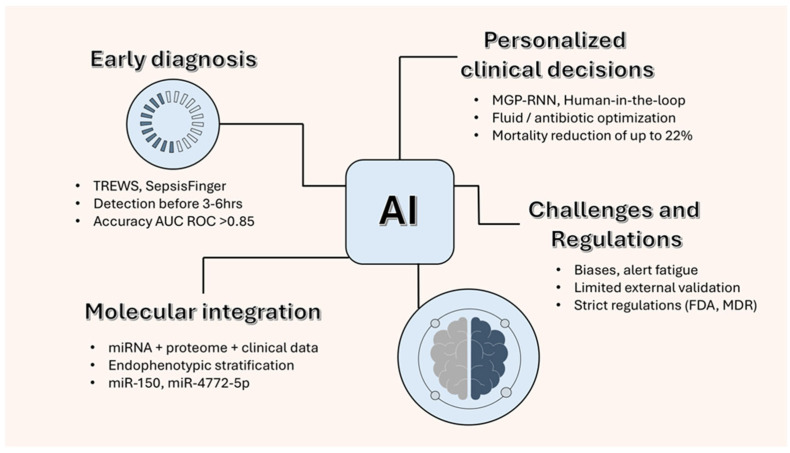
Conceptual framework integrating molecular biomarkers and Artificial Intelligence (AI) for personalized management of sepsis. The figure shows the conceptual integration of AI-driven models with molecular biomarkers like circulating *microRNAs* (*miRNAs*) and plasma gelsolin (pGSN). AI systems aid early diagnosis and personalized clinical decisions by analyzing complex clinical and biological data. The framework is still exploratory, needing multicenter validation, standardized molecular tests, and regulatory approval before it can be used clinically.

Figure 4 illustrates how integrating circulating *miRNAs* and plasma gelsolin (pGSN) data with AI-based analytic models may enhance early sepsis detection and personalized treatment decisions. This conceptual framework highlights the translational potential of combining molecular and computational approaches, while acknowledging that such integration remains at a preclinical, exploratory stage.

### 6.2. AI for Clinical Decision Support and Real-Time Treatment Monitoring

In intensive care settings, AI-assisted clinical decision-support systems are increasingly used to optimize fluid therapy, antibiotic dosing, and life-sustaining interventions. Retrospective analyses (e.g., MIMIC-III) reported reductions in mortality of up to 20% following ML-guided treatment adjustments, along with fewer prescription errors and shorter ICU stays [30,85,86].

Recent advances in multi-omics have facilitated the integration of transcriptomic, proteomic, and epigenetic data, including miRNA and pGSN signatures, into AI-driven predictive models. These multimodal frameworks can distinguish different immunological endotypes, such as hyperinflammatory and immunosuppressive phenotypes, thereby enhancing diagnostic accuracy and prognostic stratification [12,87].

Although promising, such approaches remain at a conceptual and preclinical stage, requiring multicenter validation, algorithm transparency, and reproducibility before clinical adoption.

## 7. Future Directions: AI and Biomarkers for Personalized Immunotherapy

Advances in precision medicine are transforming sepsis management by incorporating molecular biomarkers and Artificial Intelligence (AI) tools to enable personalized treatment approaches.

While microRNAs (*miRNAs*) such as *miR-155* and *miR-146a* have shown the ability to modulate inflammatory and endothelial pathways in preclinical models, their translation into therapy remains difficult due to pharmacokinetic barriers, molecular instability, and potential off-target effects [60,88].

Strategies using antagomirs or *miRNA* mimics are still in the experimental phase, needing optimized delivery methods, enhanced safety profiles, and thorough clinical testing before being used in humans.

In parallel, AI and machine learning (ML) technologies may eventually assist in personalizing immunotherapy by integrating molecular, clinical, and physiological data to predict individual treatment responses and optimize timing and dosage [89].

This integration could enable precise immunomodulation, such as identifying candidates for IL-7 supplementation, immune checkpoint inhibition (PD-1/PD-L1 blockade), or epigenetic therapies targeting dysfunctional immune states [13,90].

Nonetheless, these applications remain theoretical. Translational success will depend on multicenter validation, algorithm reproducibility, and ethical data management frameworks that promote transparency and clinical safety.

The convergence of omics biomarkers (*miRNA*, pGSN) with AI-guided analytics is an emerging yet promising approach in personalized sepsis care, where computational and biological insights could eventually support tailored, mechanism-based treatments.

## 8. Challenges in Translating AI and Biomarkers into Clinical Practice

Although the technical performance shows promise, the clinical use of AI in sepsis remains limited. Issues include selection biases, alert fatigue among doctors, data variability, and lack of external validation [91]. A prominent example is the algorithm developed by Epic Systems, which, despite heavy promotion, performed poorly in real-world settings. As reported in a 2021 study by Wong et al., the system missed approximately 67% of true sepsis cases and generated a substantial number of false alerts [92].

Meanwhile, the clinical use of *miRNAs* faces challenges due to the lack of standardization, cross-platform differences, and the need for their regulation as diagnostic tools. Incorporating a quick bedside detection panel in intensive care units, such as for miR-150 and miR-146a, could facilitate early immune profiling and personalized therapy adjustments [89,90]. For both AI and epigenetic biomarkers, the move from research to clinical practice demands a responsible, regulated, and patient-focused approach [90].

Despite encouraging findings, several practical barriers hinder the integration of molecular biomarkers and AI tools into routine sepsis care. First, the economic burden of implementing omics-based diagnostics and AI-driven platforms is significant; costs include not only those of assays but also ongoing investment in data infrastructure, cybersecurity, and technical support. This presents a challenge for low- and middle-income healthcare systems, where sepsis has the greatest impact. Second, the need for standardized assays and multicenter validation remains critical. Most candidate biomarkers, including circulating *miRNAs* and pGSN, have been studied in relatively small, diverse cohorts. Without harmonized protocols and reproducibility across different patient populations, their translation into clinical practice will remain limited.

Finally, the absence of pilot clinical models that combine biomarker-guided decision-making with AI-based tools represents a major gap. Prospective studies and pragmatic clinical trials are essential to determine whether such integrated approaches enhance patient-centered outcomes, decrease inappropriate antibiotic use, and are practical within current intensive care infrastructures.

## 9. Strengths and Limitations

This report should be considered a narrative synthesis rather than a systematic review, which may affect reproducibility. However, it is one of the first comprehensive efforts to highlight the complementary role of emerging biomarkers, such as circulating *microRNAs* and pGSN, alongside AI tools for sepsis management. Previous reviews have focused either on the diagnostic and prognostic value of *miRNAs* [13], the clinical use of AI-based models in critical care, or the application of AI-driven models in sepsis [89,91].

From a clinical perspective, circulating microRNAs (*miRNAs*) and plasma gelsolin (pGSN) may support precision strategies for managing sepsis. First, specific *miRNA* signatures (e.g., *miR-150*, *miR-146a*, *miR-122*) could help identify patients at higher risk of sepsis or organ dysfunction. Second, early changes in *miRNA* and pGSN levels may complement established clinical scores (SOFA, APACHE II) for rapid diagnosis and severity assessment. Third, sequential monitoring of these biomarkers could help evaluate treatment response and recovery. Incorporating such molecular data into AI-driven decision systems is still experimental but may ultimately improve personalized therapy and outcome prediction in critical care.

By combining these perspectives, our article uniquely emphasizes how molecular biomarkers paired with AI analytics could lead to earlier diagnosis, better immune stratification, and personalized treatment strategies in sepsis.

## 10. Conclusions

*Circulating* miRNAs and pGSN are emerging as promising biomarkers for early diagnosis, risk stratification, and outcome prediction in sepsis. Their biological relevance and consistent association with disease severity support further investigation. However, translation into clinical practice remains limited by the absence of large-scale multicenter validation, the high cost and low availability of rapid bedside assays, and the lack of standardized measurement protocols.

AI-based approaches may enhance diagnostic accuracy and facilitate personalized treatments, but their implementation requires robust digital infrastructure, interoperability across clinical systems, and external validation to ensure reproducibility and safety.

Future work should focus on multicenter prospective studies to validate biomarker panels across diverse intensive care populations and develop standardized rapid assays, and pilot trials conducted to determine whether biomarker-guided or AI-assisted strategies enhance patient-centered outcomes.

In summary, although circulating *miRNAs* and pGSN show great potential, their use as practical tools should be treated with cautious optimism, requiring validation and gradual implementation before they can enable precision medicine in sepsis.

## Figures and Tables

**Table 1:** Key features of miRNAs.

Key Features of *miRNAs* Analyzed in the Context of Sepsis	*miR-150*	*miR-146a*	*miR-155*	*miR-223*	*miR-122*	*miR-4772-5p*
Expression in Sepsis	↓	↓ (in some studies)/↑ dynamic	↑ (severe cases)	↑	↑	↑ in sepsis vs. healthy
Diagnostic Value	Not useful	Possible predictor of anti-IL-6 response	Uncertain	-	Early hepatic marker	-
Prognostic Value	↓ associated with increased mortality	Possible predictor of therapeutic response	-	Uncertain	-	-
Mechanistic Role	Inhibits NF-κB1, endothelial protection	Regulates TLR4/NF-κB via TRAF6, IRAK1	Proinflammatory, activates NF-κB	Inflammatory homeostasis regulation	Marker of subclinical liver injury	Monocyte activation, innate immune signaling
Therapeutic Potential	Promising (mimetic)	Experimental (mimetics)	Potential therapeutic target	-	-	-
Comments	Does not distinguish sepsis/non-sepsis	Dynamic role in inflammation/immunosuppression	Amplifies cytokine storm	Maintains inflammatory balance	Increases before liver test changes	Emerging marker; infection-type stratification

Legend: Comparative overview of key microRNAs (miRNAs) investigated in sepsis. This table summarizes their direction of expression, diagnostic and prognostic associations, mechanistic roles, and potential therapeutic applications. It provides a conceptual synthesis derived from multiple studies and serves as a framework for interpreting the detailed evidence presented in subsequent sections.

## Data Availability

No new data were created or analyzed in this study.

## Abbreviations

The following abbreviations are used in this manuscript:

AI	Artificial Intelligence
APACHE	Acute Physiology and Chronic Health Evaluation
ARDS	Acute Respiratory Distress Syndrome
ICU	Intensive Care Unit
IL	Interleukin
IL-1β	Interleukin 1 Beta
IL-6	Interleukin 6
LPS	Lipopolysaccharide
miRNA	microRNA
NF-κB	Nuclear Factor Kappa-light-chain-enhancer of Activated B Cells
NLRP3	NOD-like Receptor Family Pyrin Domain Containing 3
PCT	Procalcitonin
PD-1	Programmed Cell Death Protein-1
PD-L1	Programmed Death-Ligand 1
pGSN	Plasma Gelsolin
Rhu-pGSN	Recombinant Human Plasma Gelsolin
RT-qPCR	Reverse Transcription Quantitative PCR
RT-qPCR/dPCR	Reverse Transcription Quantitative Polymerase Chain Reaction/Digital PCR
SIRS	Systemic Inflammatory Response Syndrome
TLR/TLR4	Toll-Like Receptors/Toll-Like Receptor 4
VCAM1	Vascular Cell Adhesion Molecule 1
SOFA	Sequential Organ Failure Assessment
TLR4	Toll-like receptor 4
TNF-α	Tumor Necrosis Factor-α
